# Supplementary material for: A comparison of machine learning models versus clinical evaluation for mortality prediction in patients with sepsis
Source: PLoS One. 2021 Jan 19;16(1):e0245157. doi: 10.1371/journal.pone.0245157 (PMC7815112; doi:10.1371/journal.pone.0245157)
Supplement: S1 File — (DOCX) [file pone.0245157.s001.docx]

**S1 File. Extended description of clinical criteria and risk scores.**

In the current manuscript we describe several clinical criteria and risk scores. Below is a detailed description of each score or criteria and their application in our manuscript:

- **qSOFA:** the simple quick sequential organ failure assessment (qSOFA) is a bedside tool to identify patients with suspected infection who are at greater risk for a poor outcome. It uses three criteria, assigning one point for low blood pressure (systolic blood pressure ≤ 100 mmHg), high respiratory rate (≥22 breaths per min), or altered mentation (Glasgow coma scale < 15). In the current study we included patients with ≥ 2 points.
- **SIRS:** the systemic inflammatory response syndrome (SIRS) criteria has the same objective as the qSOFA criteria. SIRS assigns one point for tachycardia (heart rate > 90 beats/min), tachypnea (respiratory rate > 20 breaths/min), fever or hypothermia (temperature >38 or <36 °C), and leukocytosis, leukopenia, or bandemia (white blood cells > 12 * 10^9^/L , < 4 * 10^9^/L or bandemia ≥ 10%). In the current study we included patients with ≥2 points.
- **abbMEDS:** the abbreviated Mortality Emergency Department Sepsis (abbMEDS) score assesses sepsis severity and predicts mortality. This score assigns six points for terminal disease, three for respiratory difficulty (respiratory rate >30 breaths/min), three for septic shock, three for low thrombocytes (< 150 * 10^9^/L), three for a higher age (> 65 years), two for a lower respiratory tract infection, two for being a nursing home resident and two for an altered mental state (Glasgow coma scale < 15). abbMEDS is used in three categories: low (0 – 4 points), intermediate (5 – 12 points) and high risk (13 – 24), we used a cut-off value of 7 to dichotomize the outcome.
- **mREMS:** the modified rapid emergency medicine score (mREMS) is a triage score that is used to predict 28-day in-hospital mortality. This score assigns points for age (0: ≤44, 1: 45-64, 3: 65-74, 4: >74), systolic blood pressure (0: 110-159, 1: 160-199 and 90-109, 2: ≥200 and 80-89, 4: ≤79), heart rate (0: 70-109, 2: 110-139 and 55-69, 3: 140-179 and 40-54, 4: >179 and ≤39), respiratory rate (0: 12-24, 1: 25-34 and 10-11, 2: 6-9, 3: 35-49, 4: >49 and ≤5), oxygen saturation (0: ≥89, 1: 86-89, 3: 75-85, 4: <75) and the Glasgow coma scale (0: 14 or 15, 2: 8-13, 5: 5-7, 6: 3 or 4).
- **SOFA:** the SOFA-score was initially developed as a tool to learn from the evolution of organ failure in sepsis, but later was extensively validated to predict morbidity and mortality in several populations (Ceriani et al., 2003, Chest; Minne et al., 2008, Crit Care). It scores 1-4 points for each of the six organ systems, and we calculated it according to the formula described in the original paper (Vincent et al., 1996, Inte Care Med).
