# Supplementary material for: A comparison of machine learning models versus clinical evaluation for mortality prediction in patients with sepsis
Source: PLoS One. 2021 Jan 19;16(1):e0245157. doi: 10.1371/journal.pone.0245157 (PMC7815112; doi:10.1371/journal.pone.0245157)
Supplement: S2 File — (DOCX) [file pone.0245157.s002.docx]

**S2 File. Background information on machine learning models reviewed in the current study.**

We conducted a comparison of available algorithms on the 31-day mortality prediction task. We considered the following algorithms:

- **Logistic regression:** logistic regression is a statistical technique that in its basic form uses a logistic function to model a binary dependent variable. A simple logistic regression model was used with L2 regularization, a tolerance of 1^e-4^ and a maximum amount of iterations of 1,000. We used the limited Broyden–Fletcher–Goldfarb–Shanno (lbfgs) algorithm as optimizer. Logistic regression was implemented using Python (version 3.7.1) and the sklearn (version 0.22.1) package.
- **Multi-layer perceptron neural network:** neural networks are statistical models vaguely inspired by the biological neural networks that constitute animal brains. A simple feed-forward, multi-layer perceptron neural network was implemented consisting of three hidden layers with respectively 128, 64 and 32 neurons and ReLU activation functions. We trained the network using a constant learning rate of 0.001 with a batch size of 1 and the Adam optimization scheme. The neural network was implemented using Python programming language (version 3.7.1) using packages Keras (version 2.2.2) and scikit-learn (version 0.22.1).
- **Random Forest:** random forests is an ensemble learning method for classification that operate by constructing a multitude of decision trees at training time and outputting the class that is the mode of the classes (classification).A random forest classifier with a decision tree as base learner, consisting of 200 trees with gini criterion and a maximum depth of 50 was used. We used bootstrapped samples for building trees.
- **Gradient-boosting systems:** gradient boosting is a machine learning technique for classification problems which produce a prediction model in the form of an ensemble of weak prediction models, typically decision trees. In contrast to random forests, it builds the model in a stage-wise fashion like other boosting methods do**.** We used the XGBoost implementation of gradient-boosting systems. Each implementation has specific unique implementation details, but all use decision trees as the base weak learner and gradient boosting to iteratively fit a sequence of such trees. We used a learning rate of 0.075, a maximum number of trees of 300 and a maximum depth of each base learner to be 13. We implemented this using the Python programming language (version 3.7.1) using the package XGBoost (version 0.90).
