# Supplementary material for: A comparison of machine learning models versus clinical evaluation for mortality prediction in patients with sepsis
Source: PLoS One. 2021 Jan 19;16(1):e0245157. doi: 10.1371/journal.pone.0245157 (PMC7815112; doi:10.1371/journal.pone.0245157)
Supplement: S1 Table — The laboratory dataset consisted exclusively of laboratory variables with age, sex and time of request. The laboratory and clinical dataset contained all variables from the laboratory dataset and additionally clinical and vital characteristics. (DOCX) [file pone.0245157.s003.docx]

**S1 Table. Overview of variables present in the datasets described in the manuscript.**

The laboratory dataset consisted exclusively of laboratory variables with age, sex and time of request. The laboratory and clinical dataset contained all variables from the laboratory dataset and additionally clinical and vital characteristics.

| **Laboratory dataset^1^** | | **Laboratory and clinical dataset** |
| --- | --- | --- |
| Age  Sex  Request time  Sodium bicarbonate  ALAT  Albumin  Alkaline phosphatase  Alpha-1-fetoprotein  Ammonia  Amylase  Anti Xa  Anti-thrombin  APTT  ASAT  Atypical lymphocytes  Base excess  Basophiles  Bilirubin  Blasts  Blood transfusion  Calcium ion  Calcium total  Chloride  CK  CKD-EPI  CK-MB  Cortisol  Creatinin  CRP  D-Dimers  Direct Antiglobulin Test  Dysmorphic erytrocytes  Eosinophil  Erythroblasts  Erythrocytes  Estradiol  Ferritin  Fibrinogen  Folic acid  Fragmentocytes  PSA  Free T4  Gamma GT  Gentamycin  Glucose  Haptoglobin  HbCO  HbO2 | HDL  Hematocrit  Hemoglobin  INR  Iron  Lactate  Lactate dehydrogenase  Leukocytes  Lipase  Lymphocytes  Magnesium  MCV  MDRD  Metamyelocyte  Monocyte  Myelocyte  Neutrophils  NT-proBNP  Osmolality  pCO2  pH  Phosphate  Platelet count  pO2  Poikilocytosis  Potassium  Promyelocytes  PT  PTH  Reticulocyte  Rod-like granulocytes  Sedimentation rate  Segment core granulocytes  Sodium  Specific gravity  Standard sodium bicarbonate  Total CO2  Total protein  Toxic grain  Transferrin  Transferrin saturation  Triglycerides  Troponin T  TSH  Urea  Uric acid  Urobilinogen  Vitamin 25 (OH) D3  Vitamin B12 | Laboratory dataset variables  Weight  Length  Policy restrictions  Respiratory rate  Saturation  Temperature  Glasgow coma score  FIO2  Heart rate  Systolic BP  Diastolic BP  Inotropics/vasopressors  ECG rhythm |

^1^ For each of the laboratory variables we generated an additional binary ‘absence’ or ‘presence’ variable representing whether or not this laboratory parameter was requested.
