# Supplementary material for: A comparison of machine learning models versus clinical evaluation for mortality prediction in patients with sepsis
Source: PLoS One. 2021 Jan 19;16(1):e0245157. doi: 10.1371/journal.pone.0245157 (PMC7815112; doi:10.1371/journal.pone.0245157)
Supplement: S2 Table — We performed a baseline comparison of statistical and machine learning models (S1 File) for the 31-day mortality prediction task using the laboratory dataset. We used five-fold cross validation to assess model performance. Performance was assessed by area under the receiver operating characteristic curve (AUC) and accuracy. Confidence intervals were calculated using bootstrapping methods (n = 1,000). (DOCX) [file pone.0245157.s004.docx]

**S2 Table. Comparison of baseline statistical and machine learning models for predicting 31-day mortality risk.**

We performed a baseline comparison of statistical and machine learning models (S1 supporting information) for the 31-day mortality prediction task using the laboratory dataset. We used five-fold cross validation to assess model performance. Performance was assessed by area under the receiver operating characteristic curve (AUC) and accuracy.

| **Evaluation**  **metric** | **Logistic regression** | **Multi-layer perceptron** | **Random Forest** | **XGBoost** |
| --- | --- | --- | --- | --- |
| AUC | 0.633  (0.606 – 0.660) | 0.658  (0.632 – 0.685) | 0.723  (0.689 – 0.756) | **0.813**  **(0.791 – 0.835)** |
| Accuracy | 0.826  (0.820 – 0.833) | 0.868  (0.858 – 0.877) | 0.842  (0.831 – 0.853) | **0.873**  **(0.864 – 0.883)** |
