# Supplementary material for: A comparison of machine learning models versus clinical evaluation for mortality prediction in patients with sepsis
Source: PLoS One. 2021 Jan 19;16(1):e0245157. doi: 10.1371/journal.pone.0245157 (PMC7815112; doi:10.1371/journal.pone.0245157)
Supplement: S3 Table — Hyperparameters were based on theoretical reasoning rather than hyperparameter tuning. This was done to prevent overfitting on hyperparameters due to small sample size. “Base_score”, “Missing”, “Reg_alpha”, “Reg_lambda” and “Subsample” parameters were standard values provided by the XGBoost interface. “Max_depth”, “max_delta_step” and “estimators” were values we internally use for these kind of machine learning models. During the study, hyperparameters were never adjusted to gain performance in our validation dataset. (DOCX) [file pone.0245157.s005.docx]

**S3 Table. Hyperparameters of XGBoost models.**

Hyperparameters were based on theoretical reasoning rather than hyperparameter tuning. This was done to prevent overfitting on hyperparameters due to small sample size. “Base_score”, “Missing”, “Reg_alpha”, “Reg_lambda” and “Subsample” parameters were standard values provided by the XGBoost interface. “Max_depth”, “max_delta_step” and “estimators” were values we internally use for these kind of machine learning models. During the study, hyperparameters were never adjusted to gain performance in our validation dataset.

| **Hyperparameter** | **Value** | **Explanation** |
| --- | --- | --- |
| Max_depth | 13 | Determines how deeply each tree is allowed to grow during any boosting round. |
| Max_delta_step | 3 | Maximum delta step we allow each tree’s weight estimation to be. |
| Learning rate | 0.075 | Degree to which weights are adjusted each learning iteration. |
| Base_score | 0.5 | Initial prediction score of all instances (global bias). |
| Missing | N/A | Value which is represented as missing. Put onto N/A as no imputation was performed in data processing. |
| Reg_alpha | 0 | L1 regularization term on weights |
| Reg_lambda | 1 | L2 regularization term on weights |
| Subsample | 1 | Percentage of samples used per tree; low value can lead to underfitting. |
| Estimators | 300 | Number of trees you want to build. |
