# Supplementary material for: A comparison of machine learning models versus clinical evaluation for mortality prediction in patients with sepsis
Source: PLoS One. 2021 Jan 19;16(1):e0245157. doi: 10.1371/journal.pone.0245157 (PMC7815112; doi:10.1371/journal.pone.0245157)
Supplement: S4 Table — To study the correlation between the most important features contributing to model predictions and the clinical criteria (qSOFA and SIRS) and risk scores (abbMEDS and mREMS), we compared their existence in both. The top-20 most important features (Fig 3 in main article) are compared to all criteria in the clinical scores (S1 File). We observe that most of the features present in the clinical criteria and scores are also among the most important features in the lab and clinical machine learning model. (DOCX) [file pone.0245157.s006.docx]

**S4 Table.** **Extended analysis of correlation between important model features and clinical risk scores.**

To study the correlation between the most important features contributing to model predictions and the clinical criteria (qSOFA and SIRS) and risk scores (abbMEDS and mREMS), we compared their existence in both. The top-20 most important features (Fig. 3 in manuscript) are compared to all criteria in the clinical scores (S1 supporting information). Most of the features present in the clinical criteria and scores are also among the most important features in the lab + clinical machine learning model.

| **Lab**  **model top-20 features** | **Clinical criteria and scores** | | | | **Lab/ clinical**  **model top-20 features** | **Clinical criteria and scores** | | | |  |
| --- | --- | --- | --- | --- | --- | --- | --- | --- | --- | --- |
|  | SIRS  0/4 | qSOFA  0/3 | MEDS  2/6 | REMS  1/6 |  | SIRS  3/4 | qSOFA  3/3 | MEDS  4/6 | REMS  5/6 | |
| 1. Blood group (ordered) |  |  |  |  | 1. Heart rate | X |  |  | X | |
| 2. Urea |  |  |  |  | 2. Blood group (ordered) |  |  |  |  | |
| 3. Albumin |  |  |  |  | 3. Urea |  |  |  |  | |
| 4. CKD-EPI |  |  |  |  | 4. Albumin |  |  |  |  | |
| 5. Blood group |  |  |  |  | 5. Magnesium |  |  |  |  | |
| 6. Platelet count |  |  | X |  | 6. Platelet count |  |  | X |  | |
| 7. Age |  |  | X | X | 7. GCS |  | X | X | X | |
| 8. Total protein |  |  |  |  | 8. Oxygen saturation | X | X | X | X | |
| 9. CRP |  |  |  |  | 9. Age |  |  | X | X | |
| 10. Sodium |  |  |  |  | 10. Temperature | X |  |  |  | |
| 11. Glucose (arterial) |  |  |  |  | 11. Glucose |  |  |  |  | |
| 12. LD |  |  |  |  | 12. Systolic BP |  | X |  | X | |
| 13. Lactacte |  |  |  |  | 13. Creatinine |  |  |  |  | |
| 14. Creatinine |  |  |  |  | 14. Amount of lab |  |  |  |  | |
| 15. Lipase |  |  |  |  | 15. CRP |  |  |  |  | |
| 16. Bilirubin |  |  |  |  | 16. ALAT |  |  |  |  | |
| 17. Gamma-GT |  |  |  |  | 17. A. Fib (history) |  |  |  |  | |
| 18. Alk. phosphatase |  |  |  |  | 18. CO2 (arterial) |  |  |  |  | |
| 19. Magnesium |  |  |  |  | 19. Bilirubin |  |  |  |  | |
| 20. Hemoglobin |  |  |  |  | 20. Calcium |  |  |  |  | |
