# Supplementary material for: A comparison of machine learning models versus clinical evaluation for mortality prediction in patients with sepsis
Source: PLoS One. 2021 Jan 19;16(1):e0245157. doi: 10.1371/journal.pone.0245157 (PMC7815112; doi:10.1371/journal.pone.0245157)
Supplement: S5 Table — In addition to sensitivity and specificity, we evaluated the performance of each group by positive predictive value (PPV), negative predictive value (NPV), accuracy and area-under-the receiver operating characteristics curve (AUC). Our XGBoost model shows superior performance in each of these metrics, which is in line with the findings presented in the manuscript. (DOCX) [file pone.0245157.s007.docx]

**S5 Table.** **Extended comparison of machine learning model with internal medicine physicians and clinical risk scores.**

In addition to sensitivity and specificity, we evaluated the performance of each group by positive predictive value (PPV), negative predictive value (NPV), accuracy and area-under-the receiver operating characteristics curve (AUC). The machine learning model shows superior performance in each of these metrics, which is consistent with the findings presented in the manuscript. Confidence intervals were calculated using binomial testing and AUC’s were compared using DeLong’s test.

| **Evaluation**  **metric** | **XGBoost**  **model** | **abbMEDS** | **mREMS** | **SOFA** | **Internal medicine physicians** |
| --- | --- | --- | --- | --- | --- |
| Sensitivity, % | 92.3  (87.1 – 95.3) | 53.8  (44.1 – 63.6) | 61.5  (52.0 – 71.1) | 76.9  (68.7 – 85.2) | 72.1  (61.3 – 82.2) |
| Specificity, % | 78.2  (70.1 – 86.3) | 72.4  (63.7 – 81.2) | 64.4  (55.0 – 73.8) | 73.6  (64.9 – 82.2) | 74.2  (63.9 – 82.1) |
| PPV, % | 38.7  (29.2 – 48.3) | 22.6  (14.4 – 30.8) | 20.5  (12.6 – 28.4) | 30.3  (21.3 – 39.3) | 29.5  (20.5 – 38.4) |
| NPV, % | 98.6  (96.2 – 100.0) | 91.3  (85.8 – 96.8) | 91.8  (86.4 – 97.2) | 95.5  (91.5 – 99.6) | 94.8  (90.5 – 99.2) |
| Accuracy | 0.800  (0.722 – 0.878) | 0.700  (0.610 – 0.790) | 0.640  (0.546 – 0.734) | 0.740  (0.654 – 0.826) | 0.738  (0.651 – 0.824) |
| AUC | 0.852  (0.783 – 0.922) | 0.631  (0.537 – 0.726) | 0.630  (0.535 – 0.724) | 0.752  (0.667 – 0.836) | 0.735  (0.648 – 0.821) |
| P-value | N/A | 0.021 | 0.016 | 0.042 | 0.189; 0.072; 0.068; 0.032^a^ |

*^a^ Individual P-values were calculated for each of the internal medicine physicians.*
