# Supplementary material for: A comparison of machine learning models versus clinical evaluation for mortality prediction in patients with sepsis
Source: PLoS One. 2021 Jan 19;16(1):e0245157. doi: 10.1371/journal.pone.0245157 (PMC7815112; doi:10.1371/journal.pone.0245157)
Supplement: S6 Table — Cohen’s kappa was used to measure the inter-rater agreement between the internal medicine physicians. The level of agreement was interpreted as nil if κ was 0 to 0.20; minimal, 0.21 to 0.39; weak, 0.40 to 0.59; moderate, 0.60 to 0.79; strong, 0.80 to 0.90; and almost perfect, 0.90 to 1.3. (DOCX) [file pone.0245157.s008.docx]

**S6 Table. Inter-rater agreement of internal medicine physicians.**

Cohen’s kappa was used to measure the inter-rater agreement between the internal medicine physicians. The level of agreement was interpreted as nil if κ was 0 to 0.20; minimal, 0.21 to 0.39; weak, 0.40 to 0.59; moderate, 0.60 to 0.79; strong, 0.80 to 0.90; and almost perfect, 0.90 to 1.0.

| **Internist** | 1 (consultant) | 2 (consultant) | 3 (resident) | 4 (resident) |
| --- | --- | --- | --- | --- |
| 1 (consultant) | - | 0.52 | 0.54 | 0.46 |
| 2 (consultant) | 0.52 | - | 0.61 | 0.67 |
| 3 (resident) | 0.54 | 0.61 | - | 0.63 |
| 4 (resident) | 0.46 | 0.67 | 0.63 | - |
