# Supplementary material for: A comparison of machine learning models versus clinical evaluation for mortality prediction in patients with sepsis
Source: PLoS One. 2021 Jan 19;16(1):e0245157. doi: 10.1371/journal.pone.0245157 (PMC7815112; doi:10.1371/journal.pone.0245157)
Supplement: S7 Table — In each comparison between the machine learning model and the physicians group, a single physician was removed from the physician group. In every comparison the machine learning model outperforms the physicians. This analysis shows that the higher performance of the machine learning model was not due to systemic underperformance of a single physician. (DOCX) [file pone.0245157.s009.docx]

**S7 Table. Machine learning comparison to alternating physician groups.**

In each comparison between the machine learning model and the physicians group, a single physician was removed from the physician group. In every comparison the machine learning model outperforms the physicians. This analysis shows that the higher performance of the machine learning model was not due to systemic underperformance of a single physician.

| **Group** | **Sensitivity** | | **Specificity** | |
| --- | --- | --- | --- | --- |
|  | Mean (95% CI) | *P-value* compared to model | Mean (95% CI) | *P-value* compared to model |
| All internists | 0.72  [0.62-0.81] | <0.001 | 0.74  [0.64-0.82] | 0.509 |
| Internist 2, 3, 4 | 0.71  [0.61-0.80] | <0.001 | 0.75  [0.65-0.83] | 0.524 |
| Internist 1, 3, 4 | 0.69  [0.60-0.78] | <0.001 | 0.76  [0.67-0.84] | 0.653 |
| Internist 1, 2, 4 | 0.74  [0.66-0.83] | 0.001 | 0.74  [0.65-0.82] | 0.447 |
| Internist 1, 2, 3 | 0.77  [0.69 – 0.85] | 0.003 | 0.72  [0.63-0.81] | 0.316 |
