# Supplementary material for: A comparison of machine learning models versus clinical evaluation for mortality prediction in patients with sepsis
Source: PLoS One. 2021 Jan 19;16(1):e0245157. doi: 10.1371/journal.pone.0245157 (PMC7815112; doi:10.1371/journal.pone.0245157)
Supplement: S1 Fig — During the study period 5,967 patients that presented to our emergency department were referred to an internal medicine physician. Of these patients, 1420 patients fulfilled two or more SIRS and/or qSOFA criteria. After exclusion of 76 patients, a number of 1,344 patients were separated into development and validation datasets. (DOCX) [file pone.0245157.s010.docx]

**S1 Fig. Flow diagram of study inclusion.**

During the study period 5,967 patients that presented to our emergency department were referred to an internal medicine physician. Of these patients, 1420 patients fulfilled two or more SIRS and/or qSOFA criteria. After exclusion of 76 patients, a number of 1,344 patients were separated into development and validation datasets.

**
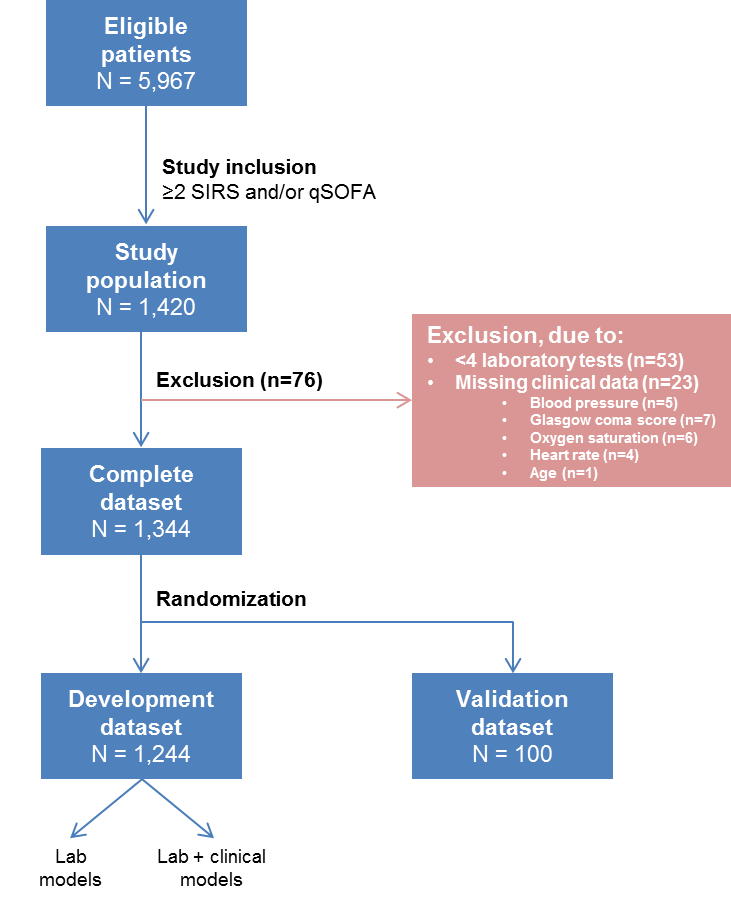
**
