# Supplementary material for: A comparison of machine learning models versus clinical evaluation for mortality prediction in patients with sepsis
Source: PLoS One. 2021 Jan 19;16(1):e0245157. doi: 10.1371/journal.pone.0245157 (PMC7815112; doi:10.1371/journal.pone.0245157)

**S2 Fig. Five-fold cross validation of diagnostic performance of XGBoost models.**

During each fold of cross-validation, we assessed predictive performance by area under the receiver operating characteristic curves (AUC). Performance was determined for models trained with laboratory data (A) and models trained with laboratory + clinical data (B) to predict 31-day mortality.


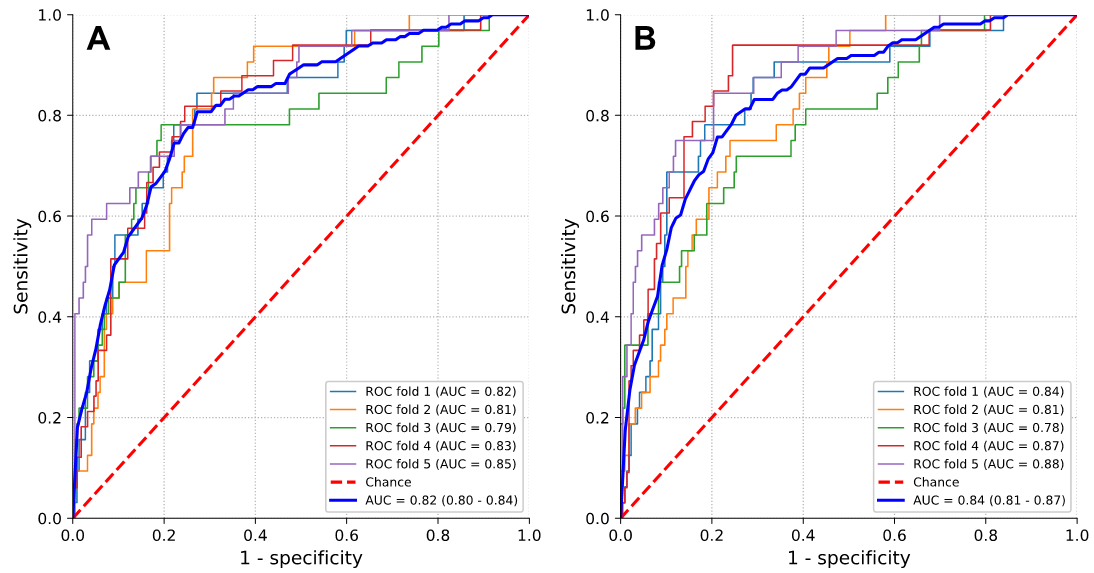

Supplement: S2 Fig — During each cycle of cross-validation, we assessed predictive performance by area under the receiver operating characteristic curves (AUC). Performance was determined for models trained with laboratory data (A) and models trained with laboratory and clinical data (B) to predict 31-day mortality. (DOCX) [file pone.0245157.s011.docx]
