# Supplementary material for: A comparison of machine learning models versus clinical evaluation for mortality prediction in patients with sepsis
Source: PLoS One. 2021 Jan 19;16(1):e0245157. doi: 10.1371/journal.pone.0245157 (PMC7815112; doi:10.1371/journal.pone.0245157)
Supplement: S3 Fig — During each cycle of cross-validation, we assessed calibration by calibration curves and their respective brier scores. Calibration was determined for models trained with laboratory data (A) and models trained with laboratory and clinical data (B). (DOCX) [file pone.0245157.s012.docx]

**S3 Fig. Five-fold cross validation of calibration of XGBoost models.**

During each fold of cross-validation, we assessed calibration by calibration curves and their respective brier scores. Calibration was determined for models trained with laboratory data (A) and models trained with laboratory + clinical data (B).

**
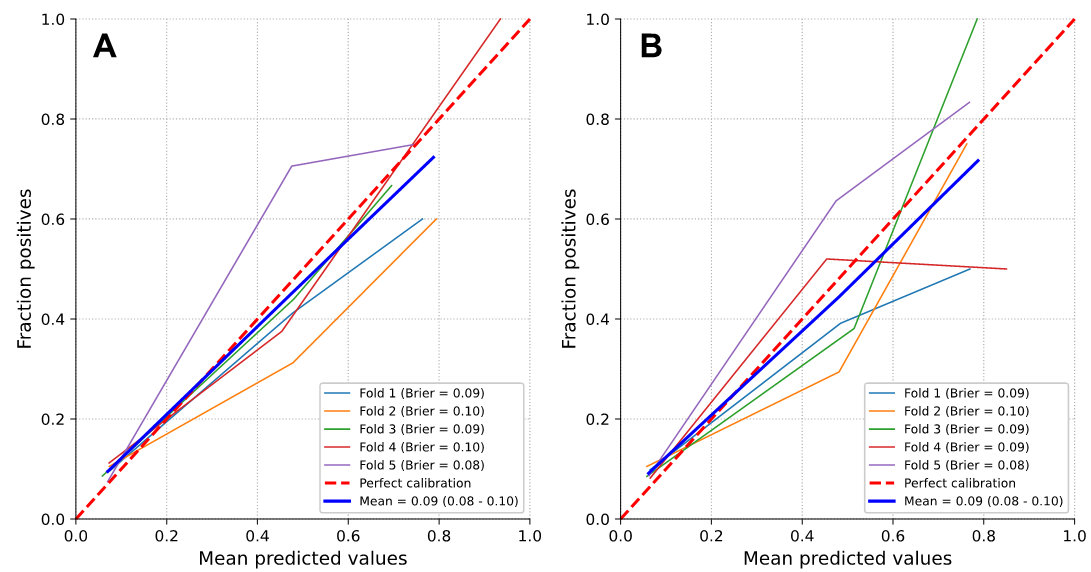
**
