# Supplementary material for: A comparison of machine learning models versus clinical evaluation for mortality prediction in patients with sepsis
Source: PLoS One. 2021 Jan 19;16(1):e0245157. doi: 10.1371/journal.pone.0245157 (PMC7815112; doi:10.1371/journal.pone.0245157)

**S4 Fig. Receiver operating characteristic analysis of machine learning model, risk scores and internal medicine physicians.**

Receiver operating characteristics analysis of the lab + clinical machine learning model (AUC: 0.852 [0.783-0.922]), abbMEDS (0.631 [0.537-0.726]), mREMS (0.630 [0.535-0.724]), SOFA (AUC: 0.752 [0.667 – 0.836]) and internal medicine physicians (mean 0.735 [0.648-0.821]). Internal medicine physicians were depicted as bullets in the ROC analysis.


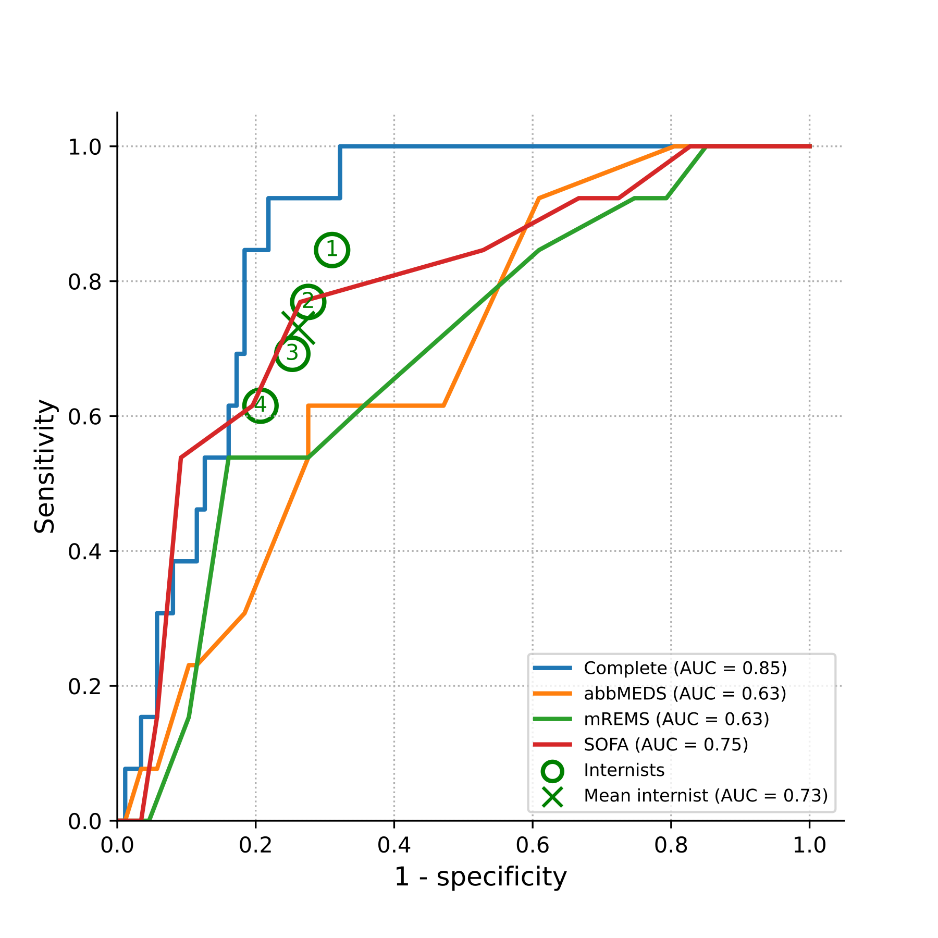

Supplement: S4 Fig — Receiver operating characteristics analysis of the lab + clinical machine learning model (AUC: 0.852 [0.783–0.922]), abbMEDS (0.631 [0.537–0.726]), mREMS (0.630 [0.535–0.724]) and internal medicine physicians (mean 0.735 [0.648–0.821]). Internal medicine physicians were depicted as bullets in the ROC analysis. (DOCX) [file pone.0245157.s013.docx]
