# Supplementary material for: A comparison of machine learning models versus clinical evaluation for mortality prediction in patients with sepsis
Source: PLoS One. 2021 Jan 19;16(1):e0245157. doi: 10.1371/journal.pone.0245157 (PMC7815112; doi:10.1371/journal.pone.0245157)
Supplement: S5 Fig — Predictive performance of all internal medicine specialists (n = 4; 2 experienced consultants in acute internal medicine and 2 experienced residents acute internal medicine) was assessed by sensitivity (left) and specificity (right). Consultants (experienced) specialists are depicted in grey and residents in orange. (DOCX) [file pone.0245157.s014.docx]

**S5 Fig. Individual performance of internal medicine physicians.**

Predictive performance of all internal medicine specialists (n=4; 2 experienced consultants in acute internal medicine and 2 experienced residents acute internal medicine) was assessed by sensitivity (left) and specificity (right). Consultants are depicted in grey and residents in orange.
