# Supplementary material for: A comparison of machine learning models versus clinical evaluation for mortality prediction in patients with sepsis
Source: PLoS One. 2021 Jan 19;16(1):e0245157. doi: 10.1371/journal.pone.0245157 (PMC7815112; doi:10.1371/journal.pone.0245157)
Supplement: Attachment — Submitted filename: Response to reviewers.pdf [file pone.0245157.s015.pdf]

Dr. Ivan Olier, PhD
Academic Editor of *PLOS ONE*
Faculty of Engineering and Technology
Liverpool John Moores University, United Kingdom

July 10, 2020

Dear Dr. Olier,

We wish to thank you for the interest in our manuscript. We appreciate the constructive comments of both reviewers which have been valuable for improving of our manuscript. Please find enclosed our revision of the manuscript “A comparison of machine learning models versus clinical evaluation for mortality prediction in patients with sepsis” (title has changed). We addressed the comments of both reviewers in a point by point rebuttal and revised our manuscript, accordingly. Track changes were used to indicate the revised sections in our manuscript. Additionally, minor textual changes were performed to improve the readability of the manuscript.

On behalf of my co-authors, I am pleased to submit a revised version of our manuscript. I confirm that this work is original and has not been published elsewhere nor is it currently under consideration for publication elsewhere. All authors have read and agreed with the revision of the manuscript. We wish to thank you for the opportunity to submit a revised version of our manuscript to PLOS ONE.

Yours sincerely,

Steven J.R. Meex, Ph.D.
Central Diagnostic Laboratory
Maastricht University Medical Center+
Post office box 5800
6202 AZ Maastricht
The Netherlands
Tel: +31 (0)43-387 4709
Fax: +31 (0)84-003 8525
E-mail: steven.meex@mumc.nl

Journal Requirements:

When submitting your revision, we need you to address these additional requirements.

1.Please ensure that your manuscript meets PLOS ONE's style requirements, including those for file naming. The PLOS ONE style templates can be found at https://journals.plos.org/plosone/s/file?id=wjVg/PLOSOne_formatting_sample_main_body.pdf andhttps://journals.plos.org/plosone/s/file?id=ba62/PLOSOne_formatting_sample_title_authors_affiliations.pdf

We adjusted the manuscript and supplemental information to meet the style requirements of PLOS ONE.

2. In your ethics statement in the manuscript and in the online submission form, please provide additional information about the patient records used in your retrospective study. Specifically, please ensure that you have discussed whether all data were fully anonymized before you accessed them and/or whether the IRB or ethics committee waived the requirement for informed consent. If patients provided informed written consent to have data from their medical records used in research, please include this information.

The data was fully anonymized before analysis. The study was approved by the medical ethical committee (METC 2019-1044) and hospital board of the Maastricht University Medical Centre+. The ethics committee waived the requirement for informed consent. We adjusted our ethics statement and methods sections in the manuscript to include this necessary information (page 7, lines 122-123).

3. Please provide additional details regarding participant consent. In the ethics statement in the Methods and online submission information, please ensure that you have specified (1) whether consent was informed and (2) what type you obtained (for instance, written or verbal, and if verbal, how it was documented and witnessed)

The ethics committee waived the requirement for informed consent and therefore we did not collect this. We adjusted our ethics statement and methods sections in the manuscript to include this necessary information (page 7, lines 122-123).

4. PLOS requires an ORCID iD for the corresponding author in Editorial Manager on papers submitted after December 6th, 2016. Please ensure that you have an ORCID iD and that it is validated in Editorial Manager. To do this, go to 'Update my Information' (in the upper left-hand corner of the main menu), and click on the Fetch/Validate link next to the ORCID field. This will take you to the ORCID site and allow you to create a new iD or authenticate a pre-existing iD in Editorial Manager. Please see the following video for instructions on linking an ORCID iD to your Editorial Manager account: https://www.youtube.com/watch?v=_xcclfuvtxQ

We added the ORCID iD for the corresponding author in the editorial manager.

5. Please ensure that you refer to Figure 4 in your text as, if accepted, production will need this reference to link the reader to the figure.

We inserted two references to Figure 4 in the last section of our results section (page 20, line 328).

6. Please include captions for your Supporting Information files at the end of your manuscript, and update any in-text citations to match accordingly. Please see our Supporting Information guidelines for more information: http://journals.plos.org/plosone/s/supporting-information.

We inserted the captions for supporting information at the end of our manuscript file and also adjusted the citations accordingly.

Reviewer 1

We are pleased with the feedback given by the reviewer. We want to thank the reviewer for evaluating our manuscript and for giving important suggestions and comments to improve our manuscript. The comments have been addressed in a point-by-point fashion in this document. The changes are highlighted in track changes throughout the manuscript. The pages and lines mentioned for each adjustment refer to the manuscript and supplemental file with track changes.

1. The manuscript presents the development of a machine learned model predicting 31-day mortality. The predictive performance of the model is compared to the predictive performance of 4 internists (2 consultants and 2 fellows). The machine learned model outperformed the internists by a wide margin. The novelty in the manuscript is the comparison between the machine learning (ML) model and the internists. Developing yet another ML model for sepsis mortality risk is not exciting; but a comparison of the model with human expert internists is exciting. As the key contribution of the paper this comparison has to be solid. Unfortunately, the way the paper stands now, I do not feel that this comparison is rock solid. Here are some weakness in the comparison.(a) Although the difference between internist and ML is high, with only 4 internists, the differences among the internists can significantly influence the results. It would be helpful to have inter-rater agreement among the internists. Also, how was specificity/sensitivity for the ML model determined?

We thank the reviewer for this in-depth analysis of our manuscript. We agree that the differences between the internists could influence the results, this is also highlighted in the individual differences in sensitivity and specificity (S4 Fig). Furthermore, we calculated the inter-rater agreement between internists using Cohen's Kappa statistics which is described in the table below. This information was included in our manuscript as S6 Table, and described in the methods (page 14, lines 254-258) and results section (page 20, lines 340-342).

**Internist**	1 (consultant)	2 (consultant)	3 (resident)	4 (resident)
1 (consultant)	-	0.52	0.54	0.46
2 (consultant)	0.52	-	0.61	0.67
3 (resident)	0.54	0.61	-	0.63
4 (resident)	0.46	0.67	0.63	-

As an additional sensitivity analysis, we compared the model to alternating physician group compositions (table below). In each comparison between the machine learning model and the physicians group, a single physician was removed from the physician group. In all comparisons the machine learning model outperforms the physicians. This information was included in our manuscript as S7 Table, and described in the methods (page 14, lines 258-260) and results sections (pages 20-21, lines 342-345). This analysis, supported by the moderate to good Cohen Kappa's, shows the higher performance of the machine learning model was not due to systemic underperformance of a single physician.

**Group**	**Sensitivity**		**Specificity**	
	Mean (95% CI)	*P-value* compared to model	Mean (95% CI)	*P-value* compared to model
All internists	0.72 [0.62-0.81]	<0.001	0.74 [0.64-0.82]	0.509
Internist 2, 3, 4	0.71 [0.61-0.80]	<0.001	0.75 [0.65-0.83]	0.524
Internist 1, 3, 4	0.69 [0.60-0.78]	<0.001	0.76 [0.67-0.84]	0.653
Internist 1, 2, 4	0.74 [0.66-0.83]	0.001	0.74 [0.65-0.82]	0.447
Internist 1, 2, 3	0.77 [0.69 – 0.85]	0.003	0.72 [0.63-0.81]	0.316

The specificity and sensitivity of the machine learning model was calculated using a standard cut-off of 0.5. We added an additional sentence to the manuscript in the methods section to clarify this: *"A machine learning prediction of higher than 0.50 was considered as a positive prediction."* (page 13, lines 228-229).

2. It should also be noted that the machine learned model incorporates physician judgement. Whether a lab test is performed or not is based on clinical judgement and is made available to the model (as the lab absence/presence indicator). A person with fewer labs have fewer problems and is hence less likely to die. How the authors handle missing values is typically completely reasonable and correct, however, in this case, it "leaks" physician knowledge to the ML model.

We agree with the reviewer that the addition of an "absence/presence" indicator also adds a certain level of physician intuition into the laboratory model. We feel however that this is not necessarily problematic from an application perspective. Noteworthy, the most important difference between the laboratory and the laboratory/clinical datasets is the explicit addition of clinical and vital characteristics such as blood pressure, heart rate and oxygen saturation. These factors are known to be important predictors of mortality, which is also observed in our SHAP analysis (Figure 3).

3. Beside the comparison, the model development itself raises some concerns. Specifically, he performance differences between the various machine learning methods is incredibly high (.63-ish for Ridge regression, .65 neural networks, .72 for random forest, but .85 for xgboost). We also typically see xgboost outperform other methods but not to this extreme extent. I am wondering whether the authors may have made some mistake evaluating xgboost. There are other indicators of a possible mistake:

- The performance of xgboost differs across different tables (.813 in Supp Tbl 2; .852 in Supp Tbl 5). With a stated confidence interval of .79-.83, .852 is quite a bit outside the confidence interval.

We share the reviewers' observation that XGBoost (and other gradient-boosting implementations) often outperform other algorithms in settings with heterogeneous, tabular datasets. As mentioned by the reviewer, we describe the baseline performance of XGBoost in S2 Table with an area-under-the ROC curve of 0.813 (0.791 – 0.835). This was done as a baseline comparison on the development variant of the laboratory dataset (n=1,244) with five-fold cross validation. This model is similar to the "lab" model presented in Figure 2, that has an AUC of 0.82 (0.80-0.84). The minor difference in AUC is most likely due to a different random initialization and different cross-validation folds being created (both models were developed in independent experiments). The XGBoost model we present in S5 Table is the best performing model from Figure 2 which is the laboratory + clinical model. This model had comparable performance with five-fold cross validation in the development dataset (AUC: 0.84, Figure 2) and in the validation dataset (AUC: 0.852, Supplementary Table 5).

To prevent any misinterpretation, we added "on the laboratory dataset" (page 10, line 177) in the manuscript and "using the laboratory dataset" (page 7, line 113) in the supplemental file.

- The learning rate is stated as .075 in Supp Tbl 5 but .001 in the text; could be a typo or actual different hyper-parameterization. A different hyper-parameterization could explain observed AUC values outside the confidence interval than random chance.

We apologize, this was a typo indeed. We corrected the learning rate of 0.001 to 0.075 in S1 supporting information in our supplemental files (page 5, line 97).

- The "95%"-confidence intervals are not 95%. In Supp Fig. 2B, only fold 1 falls consistently within the stated 95% confidence interval, the remaining 4 folds fall outside the "95%"-confidence interval for large consecutive portions of the ROC curve. How was the bootstrap estimation performed? Specifically, which data set is resampled (the development or the leave-out)?

The reviewer is correct: the 95% CI is based on the mean and standard deviation of 5 ROC calculations, each time using 4 of 5 equally sized folds of the development set. Five calculations are however insufficient to assume normality and calculate a reliable 95% CI. One slightly deviating ROC estimation can hence fall –just by statistical chance- almost completely outside the 95% CI. To prevent any confusion, we removed the 95% CI areas and calculations in S1 Fig and Fig 2 (main article) and only present the ROC's and mean of the 5 individual folds.

4. I agree with the authors that a full-fledged lattice search of the hyper-parameter space is unnecessary, but I would suggest:

- explaining how the hyper-parameters were determined (e.g. using the default values from the package)

Hyperparameter tuning is often critical in the development of high-performance machine learning models but due to the following constraints we decided not to perform any explicit hyperparameter tuning:

- Our main aim is the comparison of machine learning models versus physicians and not the development of a high-performance machine learning model which would be unrealistic given the current sample size
- It would require a third dataset to independently evaluate the different hyperparameters (e.g. a ‘tuning’/’validation’ dataset); which in our case would limit our sample size even further. An alternative would be nested cross-validation (*Parvandeh S, et al. Bioinformatics. 2020;36(10):3093-8. doi:10.1093/bioinformatics/btaa046*) but given the clinical context of the study we believe this is overly complex.

In the table below we describe the rationale for each of the chosen hyperparameters in our study. This information was added as an additional paragraph in our supplementary information in S3 Table (page 7, line 120-124).

Hyperparameter	Value	Rationale
Max_depth	13	Standard max_depth used for algorithms internally to prevent overfitting
Max_delta_step	3	Standard max_delta_step used for algorithms internally to prevent overfitting
Learning rate	0.075	Standard learning rate used for algorithms internally
Base_score	0.5	Standard value provided by the XGBoost interface
Missing	N/A	Standard value provided by the XGBoost interface
Reg_alpha	0	Standard value provided by the XGBoost interface
Reg_lambda	1	Standard value provided by the XGBoost interface
Subsample	1	Standard value provided by the XGBoost interface
Estimators	300	Standard amount of decision trees used internally for gradient-boosting systems

- explaining which if any hyper-parameters were changed (based on the CV test set; NOT the 100 patient leave-out set)

We would like to refer to the comment above; we did not adjust any hyperparameters.

- conduct a sensitivity analysis demonstrating that small changes in the hyper parameters do not lead to major perturbations in the performance.

To illustrate that small changes in hyperparameters do not lead to major pertubations in performance, we performed a grid-search with "max_depth" and "n_estimators" which are known to be amongst the most influential hyperparameters in tree-based models. We compared combinations of these hyperparameters and depicted their AUC in the table below. Models employed in our manuscript are highlighted in bold.

**Lab dataset**		**Max_depth**						
		3	5	7	9	11	13	15
**N_estimators**	100	0.819 (0.801-0.838)	0.803 (0.777-0.828)	0.813 (0.794-0.832)	0.809 (0.791-0.827)	0.803 (0.787-0.819)	0.814 (0.798-0.831)	0.811 (0.795-0.828)
	150	0.816 (0.800-0.832)	0.805 (0.783-0.828)	0.813 (0.795-0.831)	0.812 (0.796-0.828)	0.804 (0.791-0.817)	0.817 (0.804-0.829)	0.815 (0.799-0.830)
	200	0.815 (0.799-0.813)	0.804 (0.784-0.824)	0.813 (0.795-0.830)	0.810 (0.797-0.824)	0.805 (0.794-0.817)	0.815 (0.803-0.827)	0.814 (0.799-0.830)
	250	0.816 (0.799-0.832)	0.804 (0.786-0.822)	0.813 (0.796-0.829)	0.812 (0.799-0.824)	0.805 (0.794-0.816)	0.816 (0.804-0.828)	0.813 (0.799-0.828)
	300	0.814 (0.797-0.832)	0.802 (0.785-0.819)	0.811 (0.795-0.827)	0.812 (0.800-0.825)	0.806 (0.796-0.815)	**0.818 (0.802-0.840)**	0.814 (0.800-0.828)
	350	0.814 (0.797-0.832)	0.801 (0.785-0.817)	0.811 (0.796-0.826)	0.813 (0.800-0.826)	0.805 (0.796-0.814)	0.817 (0.800-0.834)	0.813 (0.800-0.826)

**Lab + clinical dataset**		**Max_depth**						
		3	5	7	9	11	13	15
**N_estimators**	100	0.838 (0.806-0.871)	0.833 (0.805-0.860)	0.830 (0.799-0.861)	0.827 (0.800-0.854)	0.826 (0.799-0.854)	0.825 (0.796-0.855)	0.827 (0.797-0.857)
	150	0.834 (0.805-0.864)	0.832 (0.807-0.857)	0.830 (0.802-0.858)	0.831 (0.804-0.858)	0.833 (0.809-0.857)	0.835 (0.808-0.862)	0.836 (0.809-0.863),
	200	0.831 (0.804-0.858)	0.830 (0.806-0.854)	0.830 (0.803-0.857)	0.831 (0.805-0.856)	0.835 (0.813-0.857)	0.836 (0.809-0.862)	0.836 (0.810-0.862)
	250	0.827 (0.801-0.852)	0.832 (0.807-0.857)	0.830 (0.802-0.857)	0.831 (0.806-0.856)	0.838 (0.816-0.860)	0.837 (0.811-0.863)	0.840 (0.813-0.868)
	300	0.824 (0.800-0.849)	0.830 (0.805-0.855)	0.829 (0.801-0.857)	0.832 (0.807-0.858)	0.838 (0.814-0.858)	**0.838 (0.811-0.865)**	0.840 (0.812-0.868)
	350	0.823 (0.799-0.848)	0.830 (0.805-0.854)	0.828 (0.800-0.856)	0.832 (0.806-0.857)	0.837 (0.814-0.860)	0.839 (0.813-0.865)	0.839 (0.811-0.867)

We would like to stress that based upon these results we cannot decide which model is better as this would require an additional dataset. Instead, these results only provide a comparison between

different hyperparameters, depicting that subtle differences in hyperparameters do not lead to major changes in performance. As the main objective of our study is to compare the machine learning models versus physicians, we would propose not to include this in the current manuscript.

5. The SHAP score is poorly explained and Figure 3 is poorly described. Readers not familiar with SHAP and violin plots will not understand this figure. An example could be useful. Eg. High values of urea (red points) impact the risk of mortality positively (large positive impact); low values reduce it slightly (modest negative impact); urea observations in most patients have no impact on mortality (the urea violin plot is widest at 0 impact).

To facilitate the interpretation of the SHAP algorithm, we added an extended description in the methods section of our paper (pages 6-7, lines 206-216) explaining the interpretation of these values and hereby hopefully facilitating the reader with enough information to interpret these values. Moreover, as the reviewer suggested, we added an example of the interpretation of SHAP values to our results section (page 18, lines 306-309).

6. A concern with xgboost is the (somewhat) black-box nature of the model. The authors address this concern by computing the SHAP score for each feature. Given that the key differentiator of trees from the other methods is their ability to detect and use interactions, SHAP may not capture this well. A better approach is to take the (say) top 20 features and build a reduced model to see the performance loss. (I picked 20 because that is what the authors focus on in Supp Tbl 4, but other numbers could illustrate their point better.)

We agree with the reviewers comment that it would be interesting to build a XGBoost model with the top-N features. We assessed the performance of XGBoost models with top-20, 15, 10, 5 and 3 features in cross-validation by AUC under the ROC curve. Results are depicted in the table below.

Laboratory dataset		Laboratory + clinical dataset	
**Top-N features**	**AUC (95% CI)**	**Top-N features**	**AUC (95% CI)**
Full	0.818 (0.802-0.840)	Full	0.838 (0.811-0.865)
20	0.808 (0.790-0.826)	20	0.827 (0.798-0.855)
15	0.801 (0.786-0.816)	15	0.816 (0.790-0.842)
10	0.772 (0.752-0.792)	10	0.792 (0.758-0.826)
5	0.728 (0.713-0.743)	5	0.759 (0.743-0.776)
3	0.693 (0.678-0.708)	3	0.710 (0.669-0.752)

The differences between the full and top-20 models are relatively small. Considering the scope of the current manuscript and the small differences, we would propose to not include this into the current manuscript.

7. Clinical significance.ML models in sepsis abound with very marginal contributions. Proposing yet another one without explaining how it can improve sepsis care is fairly meaningless. I appreciate the authors mentioning how their model could be used, and I think expanding on this is essential. The author's suggestion of using the model to identifying high risk patients (>= 50% risk of mortality) is reasonable. However, their evaluation does not quantify their contribution from this perspective. The clinical significance of this work could be significantly improved by (i) showing how much better the ML model is at identifying patients at >=50% risk (or any other high risk); (ii) clinically describing where the ML model was correct and the internists were not (again >= 50% vs

<50% can be used) ; (iii) understanding the limits of the model, clinically describing where the ML model "fails": predicts low risk of mortality while the patient dies (regardless of whether the internists made the correct prediction). Note that higher AUC does not guarantee an improved ability to identify high-risk patients; correctly ordering low-risk patients will increase the AUC yet it has not clinical significance.

We agree with the author that the development of machine learning models without any clinical application is meaningless. However, given our limited sample size, only moderate to good model performance and a main focus on the comparison of physician vs machine learning model, we believe that providing these clinical estimates is beyond the scope of the current study. However, in an ongoing follow-up study we build high-performance machine learning models (AUCs of 0.90 and higher) in four Dutch hospitals, including more than 260.000 patients. That study is more focused on the strategy towards clinical application, which would briefly be described as follows:

First, we defined the acceptable percentage of patients that are erroneously identified as “low-risk” by the algorithm (any number from 0-100%). This percentage, e.g. 1%, could be derived from an inventory of acceptable risk tolerance for adverse events by patients, health care workers, or both (*Brown TB, et al. J Emerg Med. 2010;39(2):247-52*). Then, use the corresponding negative predictive value (in this case 99%) to derive the matching algorithm prediction threshold (e.g. 0.05) and associated values for sensitivity, specificity, and proportion of subjects identified as low risk. A similar approach can be applied to identify high risk patients: define the positive predictive value that would provide an acceptable balance between true high risk patient identification and false positives, e.g. a positive predictive value of 75% would categorize x% as high-risk individuals with 1 in 4 “flaggings” by the clinical decision support tool being false positive. A higher proportion of high risk subject identification is feasible but will be at the expense of increased false positive flaggings.

8. Limitations. Some important limitation are omitted.1. The key limitation is portability. We can expect the risk scores and the internists to have similar performance if they made predictions for patients in a different health system. How about the xgboost model? Will it achieve similar performance?

We expanded on the portability of our model in our discussion section:

(i) “our sample size (especially in the area of machine learning) is relatively small, and therefore substantial further improvement of diagnostic accuracy is likely when increasing the sample size” (page 23-24, lines 406-408)

(ii) “We also limited ourselves to sepsis patients presenting to the ED, and thus it is unknown to what degree these models translate to a broader, general ED population” (page 24, lines 408-410)

(iii) “these results are based on a retrospective, single-center study which limits the external validity of our models” (page 24, lines 410-412)

9. Physicians, which actually seeing patients, may incorporate other features not captured by the EHR.

We agree with the reviewer's comment that the performance of physicians might be underestimated due to the retrospective nature of the study. We briefly discussed this limitation in our discussion, and extended this with the sentence: "As such, the performance of internal medicine physicians might be underestimated as they were not able to directly see the patient." (page 24, lines 417-419)

10. SUMMARY. The key contribution of the paper is a comparison between an ML model and human internists. There are minor flaws in the comparison (ML has access to clinical judgement). The model development process appears to have technical problems (confidence intervals appear incorrect; unknown rational for the hyper-parameterization and dependence of the performance on these parameters). The performance envelope of the model is unexplored: what clinical patient characteristics make it fail? make it perform better than the internists? Important limitations are not mentioned. I think this paper has the potential to be an influential piece of work but its contribution as it stands now is insufficient and some technical aspects may even be incorrect.

We again would like to express our gratitude for the thorough review and suggestions of the reviewer. We think that the manuscript significantly improved and hope that it now meets the standard for publication in *PLOS ONE*.

Reviewer 2

We wish to thank the reviewer for evaluating our manuscript and for providing suggestions and comments to improve our manuscript. The comments have been addressed in a point-by-point fashion in this document. The changes are highlighted in track changes throughout the manuscript. The pages and lines mentioned for each adjustment refer to the manuscript and supplemental file with track changes.

1. Mortality prediction in patients with sepsis, utilising a small dataset of patients presenting to a single emergency department. The title is a little sensational – "Machine Learning versus Physicians", and could be modified to represent the science "A comparison of machine learning models versus clinical evaluation for mortality prediction in patients with sepsis"

According to the reviewer's suggestion we changed the title to: "A comparison of machine learning models versus clinical evaluation for mortality prediction in patients with sepsis" (page 1, lines 3-4).

2. Authors testing the hypotehesis that machine learning models would outperform physician evaluation and existing clinical risk scores. Authors determined that machine learning models out performed clinicians due to higher sensitivity and specificity, however discriminatory information is not provided in the abstract.

The discriminatory ability of models versus physicians is described in S5 Table. To further emphasize this, we added this information in our abstract: *"The model had higher diagnostic accuracy with an area-under-the-ROC curve of 0.852 (95%CI: 0.783-0.922) compared to abbMEDS (0.631,0.537-0.726), mREMS (0.630,0.535-0.724) and internal medicine physicians (0.735,0.648-0.821)."* (page 4, lines 58-61).

3. The references noted in the introduction note high discriminatory scores for abbMEDS (up to 0.85), mREMS (up to 0.84) and clinician judgement (up to 0.81), suggesting similar performance to this cohort

Clinical risk scores and even clinical judgement can indeed show high discriminatory scores (up to 0.80) but vary substantially between centers. We therefore feel it is only scientifically sound to make comparisons within studies. In our study, we found AUC's of 0.631 (0.537-0.726) and 0.630 (0.535-0.724) for abbMEDS and mREMS, respectively.

4. The comment is made that "machine learning can extract information from incomplete…data" – although complexity and non-linear relationships are areas where ML does succeed, incomplete data is still a major limitation

We agree with the comment of the reviewer that incomplete missing data is still an ongoing limitation in most machine learning models. As complex and non-linear relationships are areas where machine learning currently excels, we adopted reviewer's comment and modified the sentences in our introduction section accordingly (page 6, line 93).

5. Patients with missing clinical data were excluded – how many variables needed to be missing? Were the data missing at random? A plot representing percentage of missing values would be valuable, and an analysis to confirm that the missing value distribution was equal in the training and testing set.

We included 1,420 patients in our current study of whom 76 were excluded due to missing laboratory results (n=53) or clinical data (n=23). Missing clinical data (n=23) was a result of missing blood pressure (n=5), glasgow coma score (n=7), oxygen saturation (n=6), heart rate (n=4) and age (n=1). We would like to stress that these were excluded <u>before</u> randomization of the dataset into training and testing set. Hence, similar as in a randomized controlled trail (RCT), statistical analysis after randomization to confirm equal distribution would be undesirable.

6. Similarly, the authors note that the machine learning model is capable of dealing with missing data – could further information be provided as to how GBMs deal with the issue? Further, this statement is incongruent with the previous noting that patients with missing clinical data were excluded (unless this does not refer to biomarkers, and instead to other data)

Gradient-boosting systems are models that build an ensemble of 'weaker' prediction models, mostly decision trees. Within such decision tree, each node is a test on a feature (e.g. CRP < 5 U/L or age < 70) and each branch represents an outcome for this test. During the training phase of these algorithms, we determine the features (and their cut-offs within a node) that result in the best performance given the dataset. When we specifically deal with a missing value during training, we evaluate what happens with the performance if we take one of the two leaves for the decision tree; the leaf that results in the highest performance increase on average will be defined as the "default" path to take in case there is a missing value. An extended description of this native mechanism for support of missing data in the XGBoost algorithm specifically can be found in its original paper (*Chen T, Guestrin C. XGBoost: A Scalable Tree Boosting System. arXiv:1603.02754*).

The reviewer is correct that the missing clinical data does not include the biomarkers. We adjusted the manuscript to further clarify this (page 7, line 117).

7. As biochemical markers were taken within the first two hours, and a significant proportion of treatment is performed in that time frame, was the variable of time between presentation and time of biomarker withdrawal recorded and adjusted for?

The reviewer highlights an interesting point about the time of biomarker withdrawal and its relationship to treatment in the emergency department. Unfortunately, we do not have any data on these time windows, but considering that this is evenly distributed in the training and test dataset, it is automatically captured by the model, and will not limit the performance of the models.

8. The train/test split ratio is unusual – 93:7 – can the authors explain why this was chosen? Internal validation on 100 patients is small and may affect the reproducibility of this result.

We agree with the reviewer that the train:test split ratio differs from more conventional splits of e.g. 70%/30%. However, as the main aim of the study is the comparison versus physicians, we decided

that 100 patients (which corresponds to 7%) would be a feasible amount of patients to have evaluated by our internal medicine specialists.

9. Mortality was obtained from the electronic health record – was this linked to a national health index? How were deaths out of hospital recorded in the EHR?

Mortality information was acquired through the electronic health records which are linked to municipal population registries.

10. The septic shock definition mentions a MAP ≤90 – is an alternate threshold intended here?
We thank reviewer for this comment as we intended to describe the MAP (mean arterial pressure) here but at a threshold of <65 instead of <90. This was adjusted in the manuscript accordingly (page 10, line 165).

11. The use of SHAP to explain the findings are an important addition and the authors should be credited for its inclusion.
Thank you for the comment. In response the other reviewer, we extended the description of the SHAP algorithm in the methods (pages 6-7, lines 206-216) and results section (page 18, lines 306-309).

12. Why were internal medicine physicians chosen, as opposed to emergency or intensive care physicians? Is this typical for this hospital?
We selected internal medicine physicians (n=4) that were either residents (n=2) or consultants (n=2) specialized in acute internal medicine. At the time of the study, our hospital had no emergency medicines, but rather internal medicine physicians that specialized into acute internal medicine. These physicians work at our emergency department on a day-to-day basis, and thus best suit the comparison versus a machine learning model. To clarify this we modified the manuscript at several lines to explicitly include the word "acute" into our definition of internal medicine specialists (page 13, line 224 and 232; page 20, line 324).

13. Calibration measures such as the Brier score and calibration plots should be presented for the models
We added calibration plots and brier scores in a five-fold cross validation setting of our laboratory and laboratory+clinical models in S2 Fig. This was also added to our manuscript in the methods (page 14, lines 246-247) and results section (page 17, lines 286-287).

14. There is marked class imbalance, with only 13% of the population experiencing the primary outcome of death – were oversampling methods considered?
We thank reviewer for the suggestion. In early stages we indeed considered oversampling but given the positive outcome (death) of 13%, which is reasonably high, we decided not to perform oversampling. Also, oversampling synthesizes and/or duplicates (nearly) identical positive samples from the training dataset, which can lead to potential overfitting especially at smaller sample sizes.

• **Results and Discussion**

15. There appears to be a significantly higher percentage of patients with cancer and diabetes in the training/development set

Similar to other randomization procedures (e.g. in a RCT), statistical chance may lead to slight imbalances between randomized groups. For critical parameters one can choose to distribute them evenly during randomization, but that was considered unnecessary in our study.

16. Why is the discrimination/AUC not directly compared between the ML models and the physicians and clinical scores?

The analysis of discrimination for the physicians versus models is described in S5 Table. We deliberately chose not to use discrimination (AUC under the ROC) as the main analysis in our manuscript as it might not be fully appropriate for the evaluation of the performance of internal medicine physicians. Typically, a ROC analysis shows how sensitivity (true positive rate) changes with varying specificity (true negative rate or 1 −false positive rate) for different thresholds. Internal medicine physicians, however, only provide a binary prediction (death or survive) and therefore will represented by a single point in the ROC analysis. Although this still allows calculation of an area-under-the curve, it is an ongoing debate whether or not this is a valid metric (*Muschelli J. ROC and AUC with a Binary Predictor: a Potentially Misleading Metric. Journal of Classification. 2019. doi:10.1007/s00357-019-09345-1*).

Nonetheless, we performed a ROC analysis and depicted the predictions of internal medicine physicians as bullets in the figure (see figure below). This figure is also embedded as S3 Fig in our manuscript (page 20, line 337) and supplemental files (page 12, line 172-177).

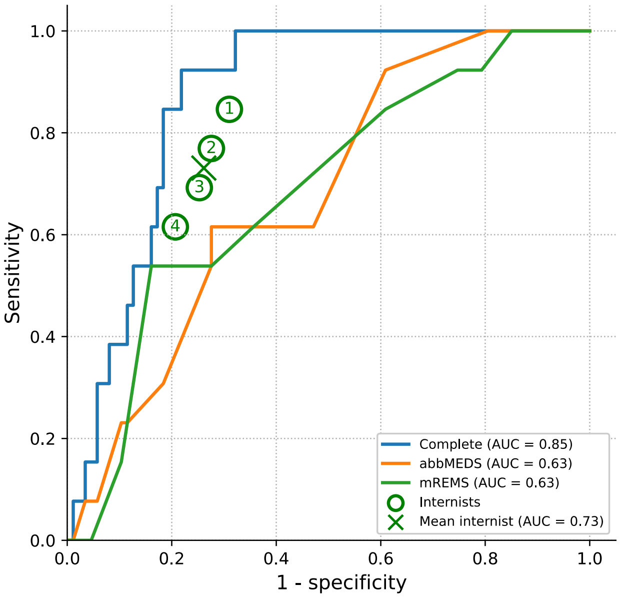

Furthermore, we recognize that the discriminatory ability of diagnostic tool is widely used and thus we decided to explicitly add a sentence regarding this comparison in our results section: "Additionally, the model had higher overall diagnostic accuracy as depicted by an AUC of 0.852 (95% CI: 0.783-0.922) compared to abbMEDS (0.631, 0.537-0.726), mREMS (0.630, 0.535-0.724) and internal medicine physicians (0.735, 0.648-0.821)." (page 20, lines 335-338).

17. AUCs should be compared using DeLong's test to demonstrate statistical superiority in regard to discrimination

We thank reviewer for the suggestion of adding DeLong's test to test for statistical significance in our ROC analysis. However, we feel that calculating these for binary predictors could be misleading. DeLong's test is mainly useful for testing differences between related predictors (e.g. an old risk score versus an updated risk score with additional variables), which is not the case in the current study.

18. Figure 2 does not include the clinical risk scores for comparison

Figure 2 shows the development of machine learning models using the development dataset. In our validation dataset of 100 patients, we provide a head-to-head comparison of the clinical risk scores with our machine learning models and internal medicine specialists (Figure 4 and S5 Table).

19. Creatinin does not share the same relationship with model output as does urea – can this be explained clinically? (from the SHAP figure)

From Figure 3 we can indeed observe that urea and creatinin have different relationships with the predictions. Urea contributes to a more positive prediction (death) especially at higher levels (indicated by the red dots), whereas creatinin mainly has a negative (protective) effect at lower biomarkers levels (indicated by blue dots). Creatinin is a marker for kidney function (and slightly for muscle mass), whereas urea is also a marker for kidney function but moreover is highly associated with hemodynamics. Hence, urea is an important marker for the overall disease state of a patient. This is also reflected in recent risk scores, such as the RISE UP, which include urea but not creatinin in their prediction models (Zelis N, Buijs J, de Leeuw PW, van Kuijk SMJ, Stassen PM. Eur J Intern Med. 2020;77:36-43.doi:10.1016/j.ejim.2020.02.021).

20. Several references to other machine learning models in the literature focused on sepsis and mortality prediction have been omitted

Based on the comment of the reviewer we conducted a new literature search and found four additional references that were included in the revised version of our manuscript:

- Horng S, Sontag DA, Halpern Y, Jernite Y, Shapiro NI, Nathanson LA. Creating an automated trigger for sepsis clinical decision support at emergency department triage using machine learning. PLoS One. 2017;12(4):e0174708. doi:10.1371/journal.pone.0174708
- Ford DW, Goodwin AJ, Simpson AN, Johnson E, Nadig N, Simpson KN. A Severe Sepsis Mortality Prediction Model and Score for Use With Administrative Data. Crit Care Med. 2016;44(2):319-27. doi:10.1097/CCM.0000000000001392
- Shukeri W, Ralib AM, Abdulah NZ, Mat-Nor MB. Sepsis mortality score for the prediction of mortality in septic patients. J Crit Care. 2018;43:163-8. doi:10.1016/j.jcrc.2017.09.009
- Bogle B, Balduino, Wolk D, Farag H, Kethireddy, Chatterjee, et al. Predicting Mortality of Sepsis Patients in a Multi-Site Healthcare System using Supervised Machine Learning2019.

21. Funded study with no conflicts of interest

Our study was funded by the non-profit organization Dutch Federation of Clinical Chemistry (NVKC). As stated in our disclosure the funders had no role in the study design, data collection, analysis, decision to publish, and preparation of the manuscript.

22. Grammar could be slightly improved however does not interfere with message of the paper
o "…and categorize from low to high risk" should be "and is categorized from low to high risk"
o "follow-studies" should be "follow-up studies"

We carefully re-read the manuscript and performed several minor modifications to improve the readability and grammar of our manuscript.

23. Authors have made data available without restriction, however note that it is available in the supplementary material – the code and raw data are not provided?

Our supplementary material presents additional information regarding the manuscript. On reasonable request the raw data and the source code used in the current study are available.
