# Supplementary material for: A comparison of machine learning models versus clinical evaluation for mortality prediction in patients with sepsis
Source: PLoS One. 2021 Jan 19;16(1):e0245157. doi: 10.1371/journal.pone.0245157 (PMC7815112; doi:10.1371/journal.pone.0245157)
Supplement: Attachment — Submitted filename: 20200901_Rebuttal - response to reviewers.pdf [file pone.0245157.s016.pdf]

Reviewer 2

We want to thank the reviewer for re-evaluating our manuscript. The comments have been addressed in a point-by-point fashion in this document. The changes are highlighted in track changes throughout the manuscript. The pages and lines mentioned for each adjustment refer to the manuscript and supplemental file with track changes.

1. The significantly lower discriminatory scores in this data compared with the literature may represent differences in the particular sample, or reflect the small sample size and thus be inaccurately portraying a lower discriminatory capacity of the physicians.

Clinical risk scores show varying discriminatory performance in the literature with area-under-the receiver operating characteristic (AUROC) ranging between 0.62-0.85 for abbMEDS and 0.62-0.84 for mREMS. The AUC in in our study for both scores is within the range reported in literature.

2. The decision to only test on 100 patients is unclear; the model may falsely appear more accurate without robust validation. Did the authors attempt validation on a randomly selected 30%?

We would like to emphasize that these 100 patients represent a random selection from the population. The validation number of 100 patients was chosen as an amount that was feasible to have carefully evaluated by 4 physicians. In regard to the reviewers suggestion to attempt validation on a randomly selected 30%, we think there may be a misunderstanding which we try to clarify: in our analysis we perform model evaluation on a randomly selected 20%, and then on top applied 5-fold cross validation, each time with another random 20% selection. We feel that our approach with 5-fold cross validation is in fact more robust than a single validation with 30%. Nevertheless, we carried out the validation suggested by the reviewer where we used a randomly selected subset of 70% to train laboratory and laboratory + clinical models, and a randomly selected 30% to evaluate the model. The AUC's (depicted below) of the resulting models are 0.84 and 0.86, which is comparable to the performance reported in our manuscript (0.82 [0.80-0.84] and 0.84 [0.81-0.87] respectively).

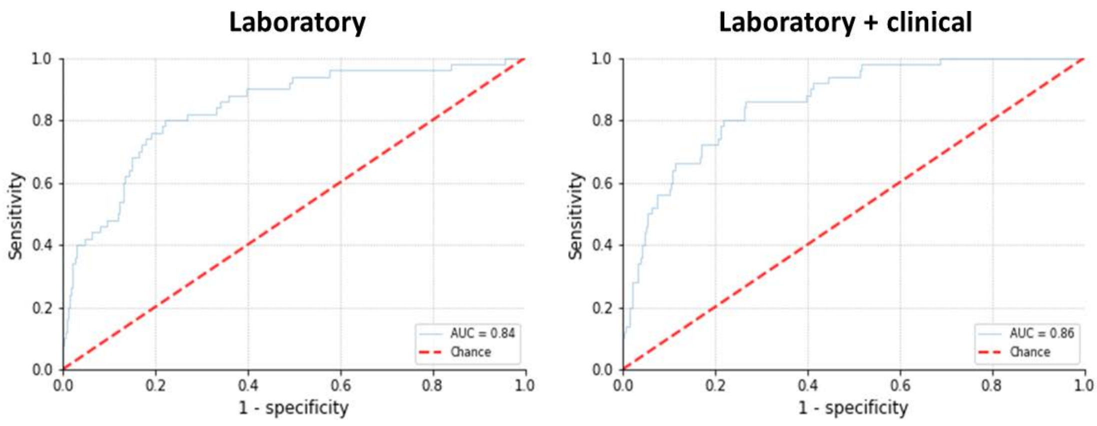

We feel that adding 70/30% resampling strategy on top of the 80/20% 5-fold cross validation to our current manuscript, would be redundant and may even be confusing to the journal's readership. We therefore propose not to include this additional analysis in the manuscript. Alternatively we will be happy to use the possibility offered by the journal to make this review correspondence publicly available alongside the article, so interested readers are able to see the additional analysis and read all considerations made during the review process.

3. Cancer and diabetes would traditionally be strongly associated with mortality, particularly in septic shock. The authors note that this was considered unnecessary to distribute these evenly, however it is unclear why.

This choice is similar to decisions in a randomization procedure in a randomized controlled trial: statistical chance may lead to slight imbalances between randomized groups (which becomes less likely with increasing sample size). If there is a single (or very limited number) of key confounding factors, one can choose to account for them and guarantee an equal distribution. In our study however, we feel that an even distribution of cancer and diabetes is not substantially more critical than many other traits: e.g. age, sex, hemodynamics at presentation, other comorbidities etc. In the absence of compelling a priori evidence to prioritize cancer and diabetes over all other potentially confounding parameters, we chose not distribute them evenly.

4. A statistical opinion should be sought regarding the direct validity of comparing AUROCs with DeLong's test; it is the reviewers opinion that this is a useful comparator for discriminatory evaluations such as this.

As suggested by the reviewer, we compared the discriminatory performance of the machine learning model versus the clinical risk scores and physicians with DeLong's test (DeLong et al, 1988) in the validation subset. The results are provided in the table below.

Model	AUC (95% CI)	P-Value
Machine learning model	0.852 (0.783 – 0.922)	N/A
abbMEDS	0.631 (0.537 – 0.726)	0.021
mREMS	0.630 (0.535 – 0.724)	0.016
Internal medicine physicians	0.735 (0.648 – 0.821)	0.189; 0.072; 0.068; 0.032[a]

[a] *Individual P-values were calculated for each of the internal medicine physicians.*

We updated the manuscript in the methods section on page 11, lines 230-231 and in the results section on page 15, lines 304-306. Additionally, we updated Supplementary Table 5 on page 8, lines 124-126 with these results.

5. The reverse relationships of urea and creatinine are unclear. Often both are not included in the same score as they are in the same direction (and consequently one will knock out the other during development); it remains unclear why they would be in opposite directions.

From a clinical perspective creatinin is a marker for kidney function (and slightly for muscle mass), whereas urea also reflects hemodynamics. Urea is therefore an important marker for the overall disease state of a patient. Although creatinin and urea are concordant in many subjects, they can differ, and reverse relationships are actually possible and should not be considered surprising. Indicative for the fact that urea and creatinin are not always concordant is the clinical use of an urea-to-creatinin ratio, which can aid in the diagnosis of prerenal injury, GI bleeding, elderly patients or hypercatabolic states (*Irwin & Rippe, 2008; Brisco et al, 2013; Sunjino et al, 2019*).
