# Supplementary material for: A comparison of machine learning models versus clinical evaluation for mortality prediction in patients with sepsis
Source: PLoS One. 2021 Jan 19;16(1):e0245157. doi: 10.1371/journal.pone.0245157 (PMC7815112; doi:10.1371/journal.pone.0245157)
Supplement: Attachment — Submitted filename: 20201105_Rebuttal - response to reviewers.docx [file pone.0245157.s017.docx]

**Reviewer #3**

**1. I have no further comments for the authors. I have read the prior comments and believe that they have been addressed in a satisfactory manner.**

We wish to thank the reviewer for evaluating our manuscript.

**Reviewer #4**

**1. Thank you for the opportunity to review this manuscript. Though interesting and seemingly sound in terms of methods, I have some concerns regarding its application (see comments below).**

We want to thank the reviewer for evaluating our manuscript and for giving important suggestions and comments to improve our manuscript. The comments have been addressed in a point-by-point fashion in this document. The changes are highlighted in track changes throughout the manuscript. The pages and lines mentioned for each adjustment refer to the manuscript and supplemental file version with track changes.

**2. In order for this study to be valid it needs to applied to an undifferentiated population with infection who could have sepsis, but do not have the diagnosis yet. As I read it, it would appear that all of the patients in this study were referred for admission for some reason, which is different from patients presenting to the ED with an infection as many of those patients would be discharged home. Therefore I don’t believe this score can be directly compared to score made for an undifferentiated ED population. However, if this is not the case, the authors should state clearly that this study included all ED data for patients meeting their SIRS/qSOFA criteria.**

This study focused on sepsis patients who visited the ED. All patients aged ≥18 years being referred to the internal medicine physician because of sepsis (i.e. a suspected or proven infection with two or more SIRS and/or qSOFA criteria) (S1 supporting information) were included in this study. This is also described in our methods section at page 6, lines 99-109. Our machine learning model was compared to the abbreviated Mortality in Emergency Department Sepsis (abbMEDS) and the modified Rapid Emergency Medicine Score (mREMS). Since the abbMEDS is specifically designed for sepsis patients, we believe this is a fair comparison. We agree with the reviewer that mREMS is a score originally developed for an undifferentiated ED population, but since it has also been validated specifically in sepsis populations (Chen et al., 2013, BMJ Emer Med J; Howell et al., 2008, Acad Emer Med; Sankoff et al., 2008, Crit Care Med; Crowe et al., 2010, J Emerg Trauma Shock), we believe it is also insightful in a comparison in our study. For completeness, and in response to your request in question 10, we also included the SOFA score in our comparison to the machine learning model and internal medicine physicians. This is described in greater detail in our response to question 10.

**3. I would be interested in knowing how this score would be applied, since the authors specifically chose to test and validate their models based on the first 2 hours of available clinical and laboratory information and from what I can tell these were all patients who were being admitted to the hospital. If this is the case it would reduce the applicability of this tool.**

We agree with the author that the development of machine learning models without any clinical application is meaningless. However, given our limited sample size, only moderate to good model performance and a main focus on the comparison of physician vs machine learning model, we believe that providing these clinical estimates is beyond the scope of the current proof-of-concept study. However, in an ongoing follow-up study we built high-performance machine learning models (AUCs of 0.90 and higher) in four Dutch hospitals, including more than 260.000 patients. That study is more focused on the strategy towards clinical application, which can be described as follows:

First, we defined the acceptable percentage of patients that are erroneously identified as “low-risk” by the algorithm (any number from 0-100%). This percentage, e.g. 1%, could be derived from an inventory of acceptable risk tolerance for adverse events by patients, health care workers, or both (*Brown TB, et al. J Emerg Med. 2010;39(2):247-52*). Then, we will use the corresponding negative predictive value (in this case 99%) to derive the matching algorithm prediction threshold (e.g. 0.05) and associated values for sensitivity, specificity, and proportion of subjects identified as low risk. A similar approach can be applied to identify high risk patients: define the positive predictive value that would provide an acceptable balance between true high risk patient identification and false positives, e.g. a positive predictive value of 75% would categorize x% as high-risk individuals with 1 in 4 “flaggings” by the clinical decision support tool being false positive. A higher proportion of high risk subject identification is feasible but will be at the expense of increased false positive flaggings.

**4. How was consent obtained on a retrospective study? How were patients able to refuse participation, since the “ethics committee waived the requirement for informed consent”**

The study was approved by the medical ethical committee (METC 2019-1044) and hospital board of the Maastricht University Medical Centre+. The ethics committee waived the requirement for informed consent. Patients who are treated in The Netherlands automatically consent for their data to be used for anonymized scientific research provided that medical ethical approval is obtained, unless the specifically refuse this.

**5. This article should have a separate stat/methods review, particularly the use of 100 patients for the validation set despite this being addressed in this revision in a 70/30 split the data in table 1 seem too sparse with several of the features having N’s in the single digits. I am not sure the standard for an ML paper. Also I am not familiar with their method for cross-validation.**

The methods and statistical analysis of our manuscript have extensively been reviewed and, after revisions, considered robust by previous reviewers (#2 and #3). Cross-validation is a model validation technique for assessing how the results of a statistical analysis will generalize to an independent data set. This was described in our methods section (page 10, lines 173-183) and is widely used and adapted in literature validating clinical prediction models (few examples include Beker, et al., 2020, Nature Mach Intell; de Rooij et al., 2020, Adv in Meth and Pract in Psych Sci; Saeb et al, 2017, Gigascience; Steyerberg et al, 2014, Eur Heart J).

**6. I am not familiar with the term acute internal medicine physicians? Do these physicians work in the ED or do they work on the acute inpatient services admitting patients?**

We selected internal medicine physicians (n=4) who were either residents (n=2) or consultants (n=2) specialized in acute internal medicine. At the time of the study, our hospital had no emergency physicians, but rather internal medicine physicians who work at our emergency department on a day-to-day basis, and thus represent the most experienced emergency medicine physicians for a comparison versus a machine learning model. They treat patients at the acute admission unit as well and are familiar with treating sepsis, infections being the main reason for ED visits they handle.

**7. How was the subpopulation of 1420 patients selected out of the 5967 patients consulted to internal medicine? Was this cohort selected randomly? There should be flow diagram showing the total populations, those excluded with reasons why and the final cohort.**

During the study period 5,967 patients that presented to our emergency department were referred to an internal medicine physician. Of these patients, 1420 patients had a suspected or proven infection and fulfilled two or more SIRS and/or qSOFA criteria. To clarify the complete process from study inclusion to data processing we depicted the flow chart below. This flow chart was also added as S1 Fig (page 10, lines 154-159) and inserted into our manuscript at page 13, line 250.

**8. The lab model vs the lab + clinical model include features that are quite different. How do the authors suggest we reconcile these differences and which model should we consider to be superior? Also please define “Blood group (present)”.**

The lab and lab + clinical model include features that are described in S1 Table. Briefly, the lab + clinical model consists of all features in the lab model with additional clinical variables. Figure 3 presents an analysis of the top-20 most important features on a model level using the SHapley Additive exPlanations (SHAP) algorithm (Lundberg et al., 2020, Nat Mach Intell). Hence, all laboratory features ranked in the top-20 lab + clinical model are also important in the lab model. The main difference is that several clinical features (e.g. heart rate, oxygen saturation and systolic blood pressure) appear to be important and therefore are ranked amongst the top-20 features.

In the current comparison, the lab + clinical model slightly outperformed the lab model (AUC 0.84 vs 0.82) and can therefore be considered superior in terms of performance. In a follow-up study (answer #3) we however decided to continue with the laboratory model as automatic, standardized collection of clinical variables is more complex and subject to variability.

We apologize for the unclear definition in relation to “Blood group (present)”. This variable represents whether or not the attending physician ordered a blood group assessment of the patient. As such this lab parameter reflects the physicians consideration to request a blood transfusion. We adjusted this in our manuscript in a revised version of Figure 3 and on page 7, lines 131-132 in the supplemental information.

**9. Some terms are not defined in the manuscript, such as GCS. Also thrombocytes should be replaced with “platelet count”.**

We thank reviewer for the suggestion. To clarify our manuscript we defined several terms in our manuscript, including platelet count (page 15, line 279 in manuscript; page 5, line 106 and page 7, lines 107 in supplementals; revised Figure 2), glasgow coma score (GCS; page 15, line 280) and C-reactive protein (CRP; page 15, line 285-286).

**10. The standard for mortality prediction in sepsis is the SOFA score. There should be a direct comparison with SOFA or at least modified version of the SOFA score (there are several versions) in order to conclude that this may have clinical utility.**

We thank the reviewer for the suggestion to include the SOFA score in the comparison we provide with the abbMEDS and mREMS clinical risk scores (Figure 4 in manuscript). We decided to implement the SOFA score in our manuscript in its original version (Vincent et al., 1996, Intensive Care Medicine) scoring 1 to 4 points for each of the six organ systems. This was included in our introduction (page 4, line 75; page 5, line 97), methods (page 10, lines 213-214), results (page 13, line 254; page 13, line 258; page 15, line 299; page 16, lines 305-308, 308-309, 312-313, 328-331), discussion (page 18, line 346) and supplemental information (page 3, lines 65-69; S5 Table; S4 Fig). The comparison with the new SOFA score was updated in a revised version of Figure 4 (depicted below).

Although it performs better than the other clinical risk scores, the machine learning models still outperforms all three clinical risk scores. We therefore believe it does not impact the major findings of our current manuscript.

**11. There needs to be more of an explanation of the different models and how they should be interpreted.**

We assume the reviewer is referring to the different machine learning models explored in the methods section of our manuscript (page 9, line 160-164). The models and their implementation details were described in S2 supporting information (page 4, lines 70-101). The results of the comparison of different models was described in S3 Table. To further clarify our models, we extended the description of our models in S2 supporting information (page 4, lines 70-101).
